# Does Patellar Resurfacing Improve Outcomes in Valgus Osteoarthritis with Compromised Patellofemoral Joint Status? A Retrospective Consecutive Comparative Study

**DOI:** 10.3390/jcm15041587

**Published:** 2026-02-18

**Authors:** Jae-Sung Seo, Jung-Kwon Bae, Seong-Kee Shin, Hyung-Gon Ryu, Kyu-Jin Kim, Ji Seon Chae

**Affiliations:** 1 Department of Orthopedic Surgery, Seoul Medical Center, Seoul 02053, Republic of Korea; 2 Department of Anesthesiology and Pain Medicine, College of Medicine, Ewha Womans University, Seoul 07804, Republic of Korea

**Keywords:** TKA, patellofemoral joint, patella resurfacing, valgus, outcomes

## Abstract

**Background/Objectives**: The benefit of patellar resurfacing (PR) in total knee arthroplasty (TKA) remains controversial. No previous study has examined the impact of PR in valgus osteoarthritis (OA) with compromised patellofemoral joint (PFJ) status. **Methods**: We retrospectively reviewed 2250 primary TKAs performed between 2011 and 2025. Among 152 valgus OA cases, 87 had compromised PFJ status, defined as Outerbridge grade 3–4 chondral damage or patellar tilt >10° on Merchant-view radiographs. Two surgeons with identical protocols operated during overlapping periods; one typically performed PR (n = 47) and the other did not (n = 40). Primary outcomes included the American Knee Society (AKS) score and Kujala Anterior Knee Pain Scale. Secondary outcomes included radiologic measures (HKA angle, patellar tilt, and lateral patella shift) and patellar-related complications (crepitus, fracture, subluxation, and maltracking). **Results**: At a mean follow-up of 7.1 years in the non-PR group and 6.5 years in the PR group, no significant differences were observed between groups in KSS function scores (non-PR 92.4 ± 3.5 vs. PR 93.0 ± 4.6, *p* = 0.54) or Kujala scores (non-PR 76.9 ± 3.5 vs. PR 77.7 ± 4.2, *p* = 0.33). Both patellar tilt and lateral patella shift showed slight postoperative reductions, but no significant difference was observed between groups (patellar tilt: non-PR 5.4° ± 0.8° vs. PR 5.7° ± 0.6°, *p* = 0.11; lateral patella shift: non-PR 2.4 ± 0.6 mm vs. PR 2.3 ± 0.7 mm, *p* = 0.75). Patellar-related complications were infrequent and showed no significant differences. **Conclusions**: Overall, PR did not demonstrate superior outcomes compared with non-PR in valgus OA patients with compromised PFJ status at mid-term follow-up.

## 1. Introduction

Patellar resurfacing (PR) during total knee arthroplasty (TKA) remains a subject of debate, particularly regarding its influence on functional outcomes and anterior knee pain in patients with end-stage arthritis [1,2,3]. Although TKA utilization has steadily increased, the decision to perform PR varies widely among surgeons and institutions due to inconclusive evidence and heterogeneous patient profiles [4,5]. Previous comparative studies have reported inconsistent results with respect to functional improvement and symptomatic relief, and consensus on the indications for PR has not been established [1,2,3,6,7]. Valgus osteoarthritis (OA), often accompanied by increased Q-angle and lateralized tracking, predisposes patients to compromised patellofemoral joint (PFJ) status, potentially resulting in persistent anterior knee symptoms [8,9]. Therefore, given the unique biomechanical environment in valgus deformity, where lateralized alignment of the extensor mechanism and increased patello–femoral contact stress are common, the effect of PR may differ from that observed in neutral or varus knees [10]. To our knowledge, no previous study has specifically evaluated the effect of PR in valgus OA with objectively compromised PFJ status, such as advanced chondral lesions or significant patellar maltracking. Accordingly, we aimed to evaluate whether PR provides measurable benefit in this subgroup of patients undergoing TKA.

We answer three questions:

(1) Does PR influence patient-reported outcomes?
(2) Does PR affect radiologic patellofemoral alignment?
(3) Do patella-related complications differ depending on whether PR is performed?

## 2. Methods

### 2.1. Study Design and Patients

**Figure 1:**
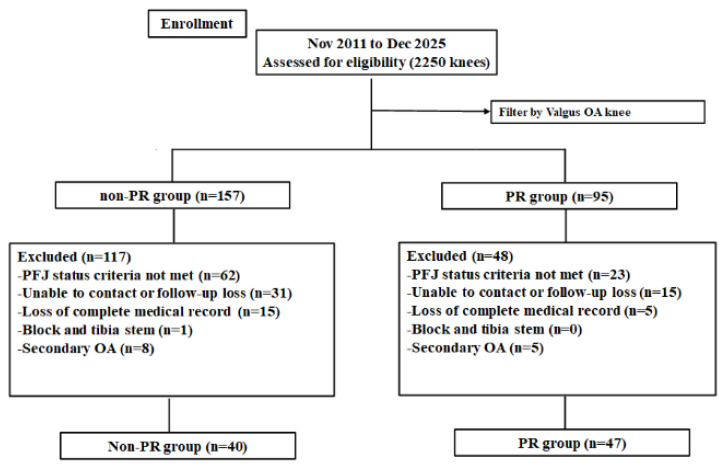
Flow diagram showing the number of knees that met the study criteria. PR, patella resurfacing.

We retrospectively collected clinical and radiologic data from consecutive patients who underwent primary TKA at our institution between January 2011 and July 2025. Among 2250 TKAs performed during this period, 152 cases involved valgus osteoarthritis (OA). Patients were screened using preoperative radiographs and intraoperative findings to evaluate patellofemoral joint (PFJ) status. Compromised PFJ status was defined as Outerbridge grade 3–4 chondral damage or patellar tilt greater than 10° on Merchant-view radiographs. Outerbridge grade 3–4 chondral damage has been widely used to indicate advanced patellofemoral cartilage degeneration, while patellar tilt greater than 10° on Merchant-view radiographs has been reported as a threshold for clinically relevant patellar maltracking [11,12]. Based on these criteria, 87 patients were eligible for inclusion. Patients were excluded if they did not meet the PFJ status criteria, were unable to be contacted or were lost to follow-up, had incomplete medical records, required tibial stem or block augmentation, or had secondary osteoarthritis. The eligible patients were assigned to either the non-patellar resurfacing (non-PR) group (n = 40) or the patella resurfacing (PR) group (n = 47) (Figure 1). Institutional review board approval was obtained (SMC 2025-11-007).

### 2.2. Surgical Techniques and Postoperative Management

All procedures were performed at a single institution by two experienced arthroplasty surgeons (SJS and BJK) who adhered to a unified operative protocol and standardized postoperative care pathway during overlapping periods. A pneumatic tourniquet was applied throughout the procedure and released after wound closure. A midline skin incision and medial parapatellar arthrotomy were used consistently. The modified gap-balancing technique was employed to achieve symmetric extension and flexion gaps [13]. Distal femoral resection was performed using an intramedullary guide targeting 3–8° of valgus alignment, and proximal tibial resection was executed with an extramedullary guide perpendicular to the mechanical axis. Femoral component rotation was determined by evaluating the balanced flexion gap, and femoral sizing was performed using an anterior reference system. All implants were cemented posterior-stabilized designs.

Patellar management followed an intraoperative decision process within the same standardized protocol. One surgeon tended to perform patellar resurfacing, whereas the other generally managed the patella without resurfacing using patelloplasty techniques. When the patella was managed without resurfacing, patelloplasty included marginal osteophyte removal, contouring of denuded facets, and circumferential electrocautery denervation [14]. Patellar tracking was evaluated throughout the range of motion using the towel clip test, and lateral release was not routinely required [15].

A closed suction drain was placed and removed within 24 h depending on wound status. Postoperative analgesia consisted of patient-controlled intravenous medication. Rehabilitation commenced on postoperative day 1, including continuous passive motion, range-of-motion exercise, quadriceps strengthening, and progressive ambulation as tolerated. Partial weight-bearing was initiated immediately and advanced to full weight-bearing over 2–3 weeks. Throughout the study period, the only variable between groups was the patellar management strategy (non-resurfacing vs. resurfacing), while all other operative and postoperative elements remained standardized.

### 2.3. Clinical and Radiologic Evaluation

Clinical outcomes were assessed using the American Knee Society score (KSS; knee and function) [16] and the Kujala Anterior Knee Pain Scale (AKP) [17], measured preoperatively and at the final follow-up. Range of motion (ROM), including flexion contracture (FC) and maximum flexion (MF), was recorded preoperatively and at each postoperative evaluation. Patella-related complications were recorded throughout the follow-up period.

Radiologic outcomes included the hip–knee–ankle (HKA) angle, patellar tilt, and lateral patellar shift, which were assessed preoperatively and at the final follow-up. The HKA angle was measured on full-length weight-bearing anteroposterior radiographs, with positive values indicating valgus alignment. Patellar tilt and lateral patellar shift were evaluated on Merchant-view radiographs. Patellar tilt [12] was defined as the angle formed between a line drawn across the widest bony margin of the patella and a reference line connecting the anterior cortical surfaces of the medial and lateral femoral condyles. Lateral patellar shift [18] was defined as the perpendicular distance from the lateral border of the patella to a reference line drawn tangentially along the anterior aspects of both femoral condyles. Radiographic measurements were independently performed and compared between the non-PR and PR groups.

### 2.4. Patella-Related Complication

Patella-related complications were recorded throughout the follow-up period. Complications included crepitus, patellar fracture, subluxation, dislocation, maltracking, and reoperation for patellar-related problems. Crepitus was defined as palpable or audible grinding during active or passive knee motion [19]. Patellar fracture was diagnosed based on clinical examination and confirmed with plain radiographs. Subluxation was defined as symptomatic lateral translation of the patella identified on physical examination and supported by radiographic assessment when indicated. Dislocation was determined by complete displacement of the patella out of the trochlear groove requiring closed or open reduction [20]. Maltracking was defined as abnormal lateral translation or tilt of the patella observed during active knee motion examination and confirmed radiographically when required [21]. Reoperation was defined as any additional surgical procedure performed to treat patellar complications.

### 2.5. Statistical Analysis

A priori power analysis was performed using G*Power software (Version 3.1.7, Universität Düsseldorf, Germany) to determine the minimum sample size required to detect a significant difference in the primary clinical outcomes (Knee Society Score and Kujala score) between groups. Assuming a power of 0.90, an alpha level of 0.05, and a standard deviation of 10 points for clinical outcome scores, a minimum of 38 patients per group was required. This assumption was based on previous TKA studies reporting standard deviations of approximately 8–12 points for Knee Society knee and function scores [22]. The present study included 40 patients in the non-PR group and 47 patients in the PR group, thereby satisfying the calculated sample size requirement.

The distribution of continuous variables was assessed using the Kolmogorov–Smirnov test. Continuous data were compared using an independent-samples *t*-test, and categorical variables were analyzed using either the Chi-square test or Fisher’s exact test where appropriate. Statistical significance was defined as a two-tailed *p*-value < 0.05. All statistical analyses were performed using SPSS software, Version 23 (IBM Corp., Armonk, NY, USA), and continuous values were expressed as mean ± standard deviation.

## 3. Results

A total of 87 patients were included in the analysis, including 40 knees in the non-patellar resurfacing (non-PR) group and 47 knees in the patellar resurfacing (PR) group. Patient demographic characteristics are summarized in Table 1. There were no significant differences between the two groups regarding sex distribution, age, BMI, ASA classification, preoperative Kellgren–Lawrence grade, preoperative HKA angle, or intraoperative cartilage status (all *p* > 0.05). Preoperative ROM showed no significant difference between the groups, including flexion contracture (*p* = 0.272) and maximal flexion (*p* = 0.296). The mean follow-up duration was 7.1 ± 2.1 years and 6.5 ± 2.8 years in the non-PR and PR groups (*p* = 0.322). The types of prosthetic components used are listed in Table 2.

**Figure 2:**
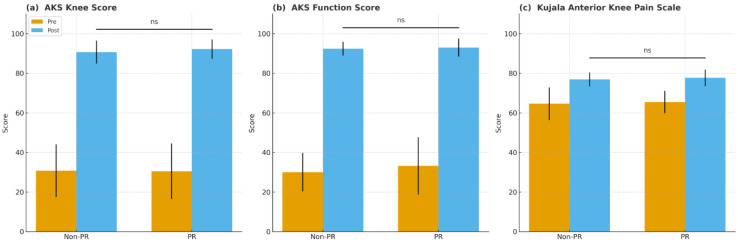
Preoperative and postoperative clinical scores in the non-resurfacing (non-PR) and patellar resurfacing (PR) groups. (**a**) American Knee Society (AKS) knee score, (**b**) American Knee Society (AKS) function score, and (**c**) Kujala Anterior Knee Pain Scale. Higher scores indicate better knee function and less anterior knee pain. Error bars represent standard deviation. ns, not significant (*p* ≥ 0.05).

Clinical outcomes showed no significant preoperative differences in AKS knee and function scores, Kujala scores, or ROM variables (all *p* > 0.05). At the final follow-up, postoperative AKS knee scores (90.7 ± 5.8 vs. 92.2 ± 4.9, *p* = 0.178), AKS function scores (92.4 ± 3.5 vs. 93.0 ± 4.6, *p* = 0.542), and Kujala scores (76.9 ± 3.5 vs. 77.7 ± 4.2, *p* = 0.328) were similar between groups. Improvements from preoperative values in AKS knee, AKS function, Kujala score, FC, and MF likewise showed no significant differences (all *p* > 0.05) (Table 3, Figure 2).

Radiologic outcomes showed no significant differences between the groups. Preoperative measurements of HKA angle, patellar tilt, and lateral patellar shift were similar (*p* = 0.226, 0.399, and 0.919, respectively), and postoperative values were also not significantly different (*p* = 0.188, 0.109, and 0.752). Changes from preoperative to postoperative assessments did not differ between groups (all *p* > 0.05) (Table 4).

Patellar-related complications, including crepitus (12.5% vs. 4.3%, *p* = 0.240), patellar fracture (0% vs. 2.1%, *p* = 1.00), dislocation/subluxation (2.5% vs. 0%, *p* = 0.460), maltracking (2.5% vs. 0%, *p* = 0.460), and reoperation (2.5% vs. 0%, *p* = 0.460), did not differ significantly between groups. The overall frequency of patellofemoral joint (PFJ) complications did not differ significantly between groups (20.0% vs. 6.4%, *p* = 0.103) (Table 5).

## 4. Discussion

The main finding of this study was that clinical and radiologic outcomes were comparable between non-patella resurfacing (non-PR) and patella resurfacing (PR) in patients with valgus OA and compromised patellofemoral joint (PFJ) status, with a mean follow-up of 7.1 years in the non-PR group and 6.5 years in the PR group. No significant differences were observed between groups in patient-reported outcomes, radiologic parameters, or patella-related complication rates.

The role of PR in primary TKA remains controversial, and previous studies have reported inconsistent findings regarding its clinical value [1,2,3]. Several reports have suggested potential benefits of patellar resurfacing for anterior knee pain and functional outcomes [2,23,24], whereas others have demonstrated no significant differences compared with non-resurfacing procedures [25,26,27]. However, these investigations have predominantly involved broad primary OA populations [1,2,3,22,23,24,25,26,27], without focusing on patients with compromised PFJ status. This study examined patients with valgus osteoarthritis, a subgroup that tends to demonstrate increased Q-angle and lateralized patellar tracking—factors that may predispose to PFJ compromises. This targeted investigation offers clinically relevant insight into a population for whom PFJ management may be particularly important, and may offer practical insight for managing TKA patients.

Previous studies evaluating Patient-Reported Outcome Measures (PROMs) have shown varied results regarding the benefit of patellar resurfacing, with some reporting improved pain relief [2,23,24] and others finding no significant differences [25,26,27]. Despite these mixed findings, our results revealed no significant differences in AKS or Kujala scores between PR and non-PR groups. One possible explanation is that functional improvement may have been sufficiently achieved with TKA alone, supported by appropriate patellar preparation—including osteophyte removal, contouring, and denervation—such that additional resurfacing did not result in a statistically detectable difference in PROMs [28,29]. Moreover, overall improvement after TKA may reduce the ability to distinguish PFJ-specific effects, and even with the Kujala score, no significant differences were identified between the groups [30].

Radiologic parameters such as patellar tilt and lateral patellar shift have shown variable postoperative changes in patellofemoral alignment after TKA [31,32,33]. In our study, both groups showed reduced patellar tilt and lateral patellar shift postoperatively, but the final values did not differ significantly between the PR and non-PR groups. This result may reflect the influence of factors such as femoral component positioning and soft-tissue balancing, including appropriate adjustment of flexion–extension gaps, on postoperative patellar tracking [34,35,36]. The comparable radiologic improvements from baseline suggest that optimized flexion–extension gap balance and accurate rotational alignment may play an important role in PFJ mechanics, potentially limiting detectable differences between groups. Moreover, performing all procedures by experienced surgeons using a standardized technique may also have reduced variability, further minimizing measurable differences in radiologic outcomes.

Patella-related complications remain an important consideration when determining the need for PR, yet previous studies have reported inconsistent findings regarding complication rates [1,37]. In our series, both individual patella-related complications—including crepitus, patellar maltracking, fracture, and reoperation—and the overall frequency of PFJ complications were low and comparable between groups, indicating that non-PR did not appear to increase patellar risk in this population. This may reflect consistent surgical technique and careful patellar preparation, which could have contributed to stable patellofemoral mechanics regardless of resurfacing status.

This study has several limitations. First, it was a retrospective study with a relatively small number of patients. The low proportion of valgus OA cases meeting the criteria for compromised PFJ status limited the feasibility of a large prospective investigation and restricted the sample size of this subgroup. In addition, a relatively large number of patients were lost to follow-up or could not be contacted, which may have introduced attrition bias. Although baseline characteristics of the included patients were comparable between the PR and non-PR groups, the exclusion of these cases may have reduced the statistical power and obscured subtle differences between groups. Despite an a priori power analysis, the limited sample size may have further constrained the ability to detect small but potentially clinically relevant differences. Second, the procedures were performed by two surgeons, and patient allocation was based on surgeon preference, which may represent a source of selection bias inherent to the retrospective design. However, to minimize this limitation, all surgeries were performed at a single institution using standardized surgical techniques and postoperative rehabilitation protocols.

In conclusion, non-patellar resurfacing (non-PR) resulted in clinical, radiologic, and patella-related complication outcomes comparable to patellar resurfacing (PR) in patients with valgus OA and compromised patellofemoral joint (PFJ) status, with a mean follow-up of 7.1 years in the non-PR group and 6.5 years in the PR group.

## Figures and Tables

**Table 1:** Patient demographics.

Variables	Non-PR Group (n = 40)	PR Group (n = 47)	*p*-Value
Sex (male/female)	3/37	5/42	0.894
Mean age (years)	73.5 ± 7.4	73.8 ± 6.7	0.863
Mean BMI (kg/m^2^)	28.0 ± 5.6	26.4 ± 4.9	0.151
Mean ASA score			0.967
1	4 (10.0)	4 (8.5)	
2	29 (72.5)	35 (74.5)	
3	7 (17.5)	8 (17.0)	
Preoperative K-L grade (3/4)	12/28	6/41	0.087
Preoperative HKA angle (°)	+6.1 ± 3.0	+7.0 ± 3.5	0.226
Preoperative ROM			
FC (°)	11.1 ± 9.4	9.0 ± 8.2	0.272
MF (°)	124.3 ± 9.4	126.1 ± 6.6	0.296
Intraoperative cartilage status (3/4)	10/30	8/39	0.516
Mean follow-up (years)	7.1 ± 2.1	6.5 ± 2.8	0.322

Values are presented as mean ± standard deviation or number (%). PR, patella resurfacing; BMI, body mass index; ASA, American Society of Anesthesiologists; K-L grade, Kellgren–Lawrence grade; HKA angle, hip–knee–ankle angle (positive values indicating valgus alignment); ROM, range of motion; FC, flexion contracture; MF, maximal flexion.

**Table 2:** The types of prosthetic component used.

	Implant	Number of Knees (%)
Non-PR group (n = 40 knees)	Vanguard^®^, Biomet (Warsaw, IN, USA)	10 (25.0)
	Genesis^®^, Smith-nephew (Watford, UK)	12 (30.0)
	Scorpio NRG^®^, Stryker (Kalamazoo, MI, USA)	8 (20.0)
	Optetrak^®^, Exactech (Gainesville, FL, USA)	6 (15.0)
	Attune^®^, DePuy Synthes (Raynham, MA, USA)	4 (10.0)
PR group (n = 47 knees)	Vanguard^®^, Biomet	9 (19.1)
	Genesis^®^, Smith-nephew	13 (27.7)
	Scorpio NRG^®^, Stryker	7 (14.9)
	Optetrak^®^, Exactech	7 (14.9)
	Attune^®^, DePuy Synthes	11 (23.4)

Values are presented as number (%). PR, patella resurfacing.

**Table 3:** Clinical outcomes between the groups.

	Non-PR Group (n = 40)	PR Group (n = 47)	*p*-Value ^a^
Preoperative			
AKS knee score	30.8 ± 13.3	30.5 ± 14.0	0.917
AKS function score	30.1 ± 9.7	33.2 ± 14.5	0.248
AKP score	64.6 ± 8.3	65.5 ± 5.6	0.576
FC (°)	11.1 ± 9.4	9.0 ± 8.2	0.272
MF (°)	124.3 ±9.4	126.1 ± 6.6	0.296
Postoperative			
AKS knee score	90.7 ± 5.8	92.2 ± 4.9	0.178
AKS function score	92.4 ± 3.5	93.0 ± 4.6	0.542
AKP score	76.9 ± 3.5	77.7 ± 4.2	0.328
FC (°)	2.1 ± 3.2	1.9 ± 3.4	0.710
MF (°)	128.5 ± 2.8	129.3 ± 1.8	0.134
Improvement from preoperative			
AKS knee score	59.9 ± 13.6	61.8 ± 14.6	0.542
AKS function score	63.7 ± 9.7	59.8 ± 15.9	0.184
AKP score	12.3 ± 8.9	12.3 ± 6.1	0.990
FC (°)	8.6 ± 6.7	7.9 ± 7.6	0.627
MF (°)	3.9 ± 6.6	3.4 ± 5.9	0.725

Values are presented as mean ± standard deviation. PR, patella resurfacing; AKS, American Knee Society knee score; AKP, Kujala Anterior Knee Pain Scale; FC, flexion contracture; MF, maximal flexion. ^a^ Student’s *t*-test was used to compare continuous variable outcomes between groups.

**Table 4:** Radiologic outcomes between the groups.

	Non-PR Group (n = 40)	PR Group (n = 47)	*p*-Value ^a^
Preoperative			
HKA angle (°)	+6.1 ± 3.0	+7.0 ± 3.5	0.226
Patella tilt (°)	6.3 ± 1.2	6.5 ± 0.7	0.399
Lateral patella shift (mm)	2.7 ± 0.7	2.7 ± 0.8	0.919
Postoperative			
HKA angle (°)	+1.7 ± 1.2	+1.4 ± 1.0	0.188
Patella tilt (°)	5.4 ± 0.8	5.7 ± 0.6	0.109
Lateral patella shift (mm)	2.4 ± 0.6	2.3 ± 0.7	0.752
Improvement from preoperative			
HKA angle (°)	+4.4 ± 3.0	+5.6 ± 3.1	0.082
Patella tilt (°)	0.9 ± 1.4	0.8 ± 0.9	0.806
Lateral patella shift (mm)	0.3 ± 0.1	0.3 ± 0.3	0.255

Values are presented as mean ± standard deviation. PR, patella resurfacing; HKA angle, hip–knee–ankle angle (positive values indicating valgus alignment). ^a^ Student’s *t*-test was used to compare continuous variable outcomes between groups.

**Table 5:** Comparison of complications between the groups.

Variables	Non-PR Group (n = 40)	PR Group (n = 47)	*p*-Value ^a^
Crepitus	5 (12.5)	2 (4.3)	0.240
Fracture	0 (0)	1 (2.1)	1
Dislocation and Subluxation	1 (2.5)	0 (0)	0.460
Patella Maltracking	1 (2.5)	0 (0)	0.460
Reoperation	1 (2.5)	0 (0)	0.460
Overall PFJ Complications	8 (20)	3 (6.4)	0.103

Values are presented as number (%). PR, patella resurfacing; PFJ, patellofemoral joint. ^a^ Fisher’s exact test.

## Data Availability

The raw data supporting the conclusions of this article will be made available by the authors on request.
